# Current distribution and voltinism of the brown marmorated stink bug, *Halyomorpha halys*, in Switzerland and its response to climate change using a high-resolution CLIMEX model

**DOI:** 10.1007/s00484-020-01992-z

**Published:** 2020-08-28

**Authors:** Sibylle Stoeckli, Raphael Felber, Tim Haye

**Affiliations:** 1 Research Institute of Organic Agriculture (FiBL), Ackerstrasse 113, P.O. Box 219, 5070 Frick, Switzerland; 2 Agroscope, Reckenholzstrasse 191, 8046 Zurich, Switzerland; 3 Now at: Office for Environment, Canton of Zug, Aabachstrasse 5, 6300 Zug, Switzerland; 4 CABI, Rue des Grillons 1, 2800 Delémont, Switzerland

**Keywords:** CLIMEX, Climate change scenarios, Localised climate data, Climate impact models, Invasive species

## Abstract

Climate change can alter the habitat suitability of invasive species and promote their establishment. The highly polyphagous brown marmorated stinkbug, *Halyomorpha halys* Stål (Hemiptera: Pentatomidae), is native to East Asia and invasive in Europe and North America, damaging a wide variety of fruit and vegetable crops. In Switzerland, crop damage and increasing populations have been observed since 2017 and related to increasing temperatures. We studied the climatic suitability, population growth, and the number of generations under present and future climate conditions for *H. halys* in Switzerland, using a modified version of the bioclimatic model package CLIMEX. To address the high topographic variability in Switzerland, model simulations were based on climate data of high spatial resolution (approx. 2 km), which significantly increased their explanatory power, and identified many more climatically suitable areas in comparison to previous models. The validation of the CLIMEX model using observational records collected in a citizen science initiative between 2004 and 2019 revealed that more than 15 years after its accidental introduction, *H. halys* has colonised nearly all bioclimatic suitable areas in Switzerland and there is limited potential for range expansion into new areas under present climate conditions. Simulations with climate change scenarios suggest an extensive range expansion into higher altitudes, an increase in generations per year, an earlier start of *H. halys* activity in spring and a prolonged period for nymphs to complete development in autumn. A permanent shift from one to two generations per year and the associated population growth of *H. halys* may result in increasing crop damages in Switzerland. These results highlight the need for monitoring the spread and population development in the north-western part of Switzerland and higher altitudes of the valleys of the south.

## Introduction

Agricultural losses from insect pests are estimated up to 20–50% (Oerke 2006; Yudelman et al. 1998) and are projected to increase under future climate conditions (Battisti and Naylor 2009). Ectotherms are especially sensitive to an increase in temperature as their basic functions strongly depend on the environmental temperature (Deutsch et al. 2018). There is strong evidence that climate change is already modifying species geographical range, phenology, voltinism and interactions (Altermatt 2010; Bebber et al. 2013; Devictor et al. 2012; Hickling et al. 2006; Maclean and Wilson 2011; Parmesan and Yohe 2003; Robinet and Roques 2010; Roth et al. 2014; Walther et al. 2002). The type and strength of the effect of climate change on species depends on taxonomic group or location. The potential effect of future climate change on insect pests based on different emission levels and regional climate scenarios has been quantified in various studies (Caffarra et al. 2012; Harrington et al. 2007; Jonsson et al. 2009; Kistner 2017; Kocmankova et al. 2011; Langille et al. 2017; Meynard et al. 2013; Olfert et al. 2016; Porter et al. 1991; Stoeckli et al. 2012; Thomson et al. 2010; Trnka et al. 2007; Warren et al. 2018; Ziter et al. 2012). These projections indicate changes in distributions with expansions northward and uphill and contractions southward and downhill. Estimated changes in seasonal phenology include earlier spring occurrences, extended flight periods, and a change from uni-voltinism to multi-voltinism. Decreased winter mortality may also lead to higher population densities early in the year. Changes in species interactions (e.g. insect-host plant, host-parasitoid, competition or decoupling of mutualism) are realistic scenarios (Both et al. 2009; Gutierrez et al. 2010; Hance et al. 2007; Tylianakis et al. 2008). Climate change comprises various abiotic stressors, such as warmer temperatures, elevated CO_2_, more frequent droughts or storms leading to complex interactions with insect pests (Jactel et al. 2019).

Besides a shift in the distribution and phenology of native pest species, it is evident that the number of non-native species will increase and that climate change will promote their establishment (Bacon et al. 2013; Seebens et al. 2017). The brown marmorated stinkbug, *Halyomorpha halys* (Stål; Hemiptera: Pentatomidae) is native to East Asia (China, Japan, Korea and Taiwan) and is a highly polyphagous pest with more than 200 wild and cultivated host plants, mainly fruit trees in the family Rosacea, but also vegetables, berries, vine, maize or soya (Lee et al. 2013). *Halyomorpha halys* invaded North America in the mid-1990s (Hoebeke and Carter 2003), where it soon became a major pest in apples, peaches, sweet corn and beans in the Mid-Atlantic region (Leskey et al. 2012). The oldest photographic evidence for the presence of *H. halys* in Europe is from Zurich, Switzerland, in 2004 (Haye et al. 2015), and since then it has established in many European countries (Cianferoni et al. 2018) and continues its spread throughout Eurasia (Tytar and Kozynenko 2020). Where the pest is bivoltine, severe damage has been observed in less than 5 years after establishment, e.g. Italy and the Republic of Georgia (Bosco et al. 2018; Maistrello et al. 2017).

Initially, the natural spread of *H. halys* in Switzerland was relatively slow, and populations were mostly restricted to the cities of Zurich and Basel. Until recently, *H. halys* has mainly been considered an urban nuisance pest. However, since 2015, significant damage in peach and pear orchards has been reported in the south, and since 2017, high population densities and damage were also noticed in fruit orchards in north-eastern Switzerland. In northern Switzerland, *H. halys* is ostensibly univoltine (Haye et al. 2014). However, in recent years (2018–2019), two generations have been reported (Haye, personal observation), and it is assumed that two generations are only possible in warmer years. In the warmer climate of northern Italy, *H. halys* appears to be bivoltine (Costi et al. 2017).

Observations and projections of the impact of climate change on environment, industry and society are highly relevant to decisions and adaptation measures of governments, political and business sectors and the society (EEA 2017; Hofmann et al. 2011; Wilby et al. 2009). Regional impact initiatives, which are of high importance for developing adaptation measures, require localised understanding of how climate change affects natural and human systems (CH2014-Impacts 2014; Henne et al. 2018). Different modelling approaches have been used to estimate the response of native and non-native insects to climate change (Hill and Thomson 2015). One of them, CLIMEX, is a semi-mechanistic bioclimatic model package with proven reliability to explore the impact of climate on the potential distribution and seasonality of invasive species (Kriticos et al. 2015b). This model has some similarities with simple correlative models (Felber et al. 2018), but it simulates the mechanisms that limit species’ geographical distributions and determines their seasonal phenology. CLIMEX models have been developed primarily for risk assessment of invasive pests (Byeon et al. 2018; de Villiers et al. 2016; Kistner-Thomas 2019; Kriticos et al. 2015c; Olfert et al. 2016; Ramos et al. 2019; Vera et al. 2002; Yonow et al. 2017).

CLIMEX models are often run on a spatial resolution of 0.16° (18.5 km) or 0.5° (55.5 km) climate data (Kriticos et al. 2012). However, this low spatial resolution does not allow adequate pest risk analyses for countries like Switzerland that have a complex topographic structure (Kriticos et al. 2017). Although *H. halys* is fairly widespread and abundant, most regions of Switzerland have been considered climatically unsuitable or only marginally suitable for *H. halys* in previous CLIMEX models run using the CliMond Climate Dataset (Kistner 2017; Kriticos et al. 2017). Accordingly, in the present study, a new CLIMEX version was developed to run with climate data of higher spatial resolution. Using this adapted CLIMEX model, the aim of this study was to re-examine the climatic suitability, population growth, the number of *H. halys* generations per year and the influence of different stress indices (e.g. cold, heat) under present and future climate conditions in Switzerland. To further validate the new fine-scale model, we were particularly interested in whether the model projections would actually match with long-term distribution records collected in a citizen science initiative in Switzerland. Finally, we aimed to identify highly suitable regions that have not yet been colonised by *H. halys* and those regions that may suffer from increasing *H. halys* populations in the near future in order to adjust current monitoring programmes and develop sustainable management strategies.

## Materials and methods

### Bioclimatic modelling with CLIMEX

CLIMEX is a semi-mechanistic bioclimatic modelling package that can estimate potential, climatically suitable areas and population dynamics for poikilothermic organisms (Kriticos et al. 2015b). It combines weekly growth and stress functions to calculate the annual Ecoclimatic Index (EI), as an estimate of the annual climatic suitability of the location for a given species. EI values <1 indicate that a species cannot survive at the location. The weekly (GI_W_) and annual (GI_A_) Growth Indices are a function of temperature (TI) and soil moisture (MI) indices. Different stress indices as cold (CS), dry (DS), heat (HS) and wet (WS) stress and their interactions are considered to simulate the mechanisms that limit survival during unfavourable seasons. Furthermore, the minimum length of the growing season (PDD) and obligate diapause can constrain the overall climate suitability. The diapause Index (DI) is based on temperature and day length. The CLIMEX model for *H. halys* developed by Kriticos et al. (2017) was used to evaluate the potential distribution and population dynamics of this species in Switzerland (Table 1).

**Table 1:** CLIMEX parameter values fitted for *Halyomorpha halys* based on Kriticos et al. (2017). Values without units are dimensionless indices

Parameter name	Abbreviation	Value
*Temperature*		
Limiting low temperature	DVO	12 °C
Lower optimal temperature	DV1	27 °C
Upper optimal temperature	DV2	30 °C
Limiting high temperature	DV3	33 °C
Degree days per generation	PDD	595 °C days
*Soil moisture*		
Limiting low soil moisture	SM0	0.1
Lower optimal soil moisture	SM1	0.5
Upper optimal soil moisture	SM2	1
Limiting high soil moisture	SM3	1.5
*Diapause*		
Diapause induction day length	DPD0	12 h light
Diapause induction temperature	DPT0	5 °C
Diapause termination temperature	DPT1	5 °C
Diapause development days	DPD	0 days
Diapause summer (1) or winter(0)	DPSW	0
*Cold stress*		
Temperature threshold	TTCS	−18 °C
Stress accumulation rate	THCS	−0.01 week^−1^
*Heat stress*		
Temperature threshold	TTHS	33 °C
Stress accumulation rate	THHS	0.01 week^−1^
*Dry stress*		
Threshold soil moisture	SMDS	0.1
Stress accumulation rate	HDS	−0.01 week^−1^
*Wet stress*		
Threshold soil moisture	SMWS	1.5
Stress accumulation rate	HWS	0.002 week^−1^
*Hot-wet stress*		
Threshold soil moisture	TTHW	28
Threshold temperature	MTHW	1.5
Stress accumulation rate	PHW	0.007 week^−1^

### Spatial climate data and scenarios for Switzerland

Projecting present and future climate for regions characterised by complex topography is challenging, and substantial spatial variation is expected, especially for precipitation patterns (Jasper et al. 2004). The original *H. halys* CLIMEX model was calibrated with distribution records from Asia and validated with records from the invaded areas of the USA and Europe using a spatial resolution of 0.16° (18.5 km) (Kriticos et al. 2017). A new CLIMEX version (4.1) was recently developed in collaboration with CSIRO (Darren Kriticos, unpublished), to allow importing climate data of higher spatial resolution. Monthly average of daily minimum and maximum temperatures 2 m above ground level and the monthly precipitation sum interpolated from observed data at a regular latitude-longitude grid of 0.02° (2.2 km) were provided by MeteoSwiss. In total, we included 11,211 grid cells. Relative humidity at 9 am and 3 pm was deduced from the saturated vapour pressure at the corresponding air temperature and averaged at the monthly level (Kriticos et al. 2012).

CH2011 Extension Series provides gridded change signals of near-surface temperature at 2 m above ground and precipitation for the two non-intervention emission scenarios A1B, A2, and the climate stabilisation scenario RCP3PD (CH2011 2011; Zubler et al. 2014). These data cover the mean annual cycle for the periods 2020–2049, 2045–2074, and 2070–2099. Scenario data were created by adding the change signal to the monthly baseline data of the reference period (1981–2010). The scenario data were used to simulate the potential future distribution of *H. halys.* The same localised interpolated climate data set was used to analyse the climate suitability for *H. halys* at 10 Swiss locations. We selected the grid cell in which the locations falls based on longitude and latitude.

### Validation of the CLIMEX model with observation records from Switzerland

The present distribution of *H. halys* in Switzerland was determined with a citizen science approach, collecting voluntary observation reports between 2012 and 2019 from private Swiss households (www.halyomorphahalys.com). All observations of eggs, nymphs or adults were confirmed by Haye with the help of photos submitted with the observations. In addition, published records of *H. halys* in Switzerland prior to 2012 (Wermelinger et al. 2007; Wyniger and Kment 2010) were added to the database. In total, the dataset consisted of 655 entries, with a continuous increase in observations each year. Model validation was conducted graphically comparing the Ecoclimatic Index (EI) for individual years (2015–2019) and Swiss observation records of *H. halys* in corresponding years. In addition, the model was validated using meteorological data for the reference period 1981–2010 against all observation records combined (i.e. 2004–2019)*.* Furthermore, the percentage of (highly) suitable areas and the percentage of areas with one or more generations was determined for each of the ten Swiss locations listed in Table 2.

**Table 2:** Percentage of (highly) suitable areas for *H. halys* in Switzerland (Ecoclimatic Index EI > 5, EI > 15) and percentage of areas producing one or two generations per year. The percentage is based on the total number cells (2 km resolution) including all alpine areas (*n* = 11211). The simulation for the reference period (1981–2010) and individual years (2015–2019) and the three climate scenarios RCP3PD, A1B and A2 (2020–2049, 2045–2074, 2070–2099) are presented

	% of suitable area (EI > 5)	% of highly suitable area (EI > 15)	% area one generation or more	% area two generations or more
Reference period				
1981–2010	23.0	0.3	33.2	0.3
Individual years				
2015	43.9	0.7	44.6	1.1
2016	31.3	0.1	38.4	0.7
2017	37.9	4.0	43.1	1.1
2018	49.1	8.4	50.1	6.0
2019	39.5	3.2	43.0	1.0
RCP3PD scenario				
2020–2049	32.1	4.9	43.1	1.0
2045–2074	36.6	8.3	45.3	1.5
2070–2099	37.3	8.7	45.5	1.6
A1B scenario				
2020–2049	32.6	5.2	43.7	1.1
2045–2074	43.5	20.5	52.3	7.5
2070–2099	52.3	32.5	59.6	26.7
A2 scenario				
2020–2049	41.8	4.5	42.6	0.9
2045–2074	52.1	19.9	51.9	7.0
2070–2099	63.6	36.9	63.5	34.8

### Potential distribution, phenology and voltinism under present and future climate conditions in Switzerland

The potential distribution of *H. halys* under present and future climate was evaluated based on the Ecoclimatic Index (EI). We termed a location with an EI > 5 as climatically suitable and a location with an EI > 15 as climatically highly suitable. The shift in the weekly Growth Index (GI_W_) was analysed to evaluate the impact of climate change on the phenology. The number of weeks with a GI_W_ > 0 was calculated as a measure for how long *H. halys* is able to grow under present and future climate conditions. The number of *H. halys* generations per year was simulated for present and future climate conditions based on a value of 595 degree days above 12 °C (Kriticos et al. 2017).

## Results

### Validation of the CLIMEX model with observation records from Switzerland

**Fig. 1:**
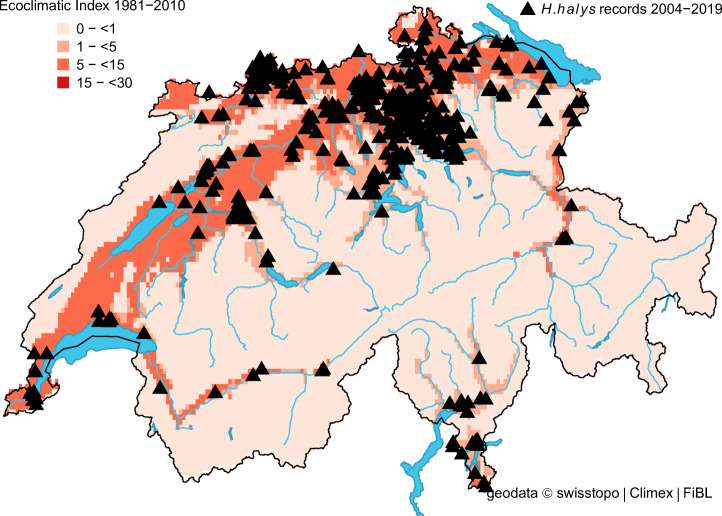
Modelled climate suitability (CLIMEX Ecoclimatic Index EI) for *Halyomorpha halys* in Switzerland for the reference period 1981–2010. Black triangles represent observation records from 2004 to 2019

**Fig. 2:**
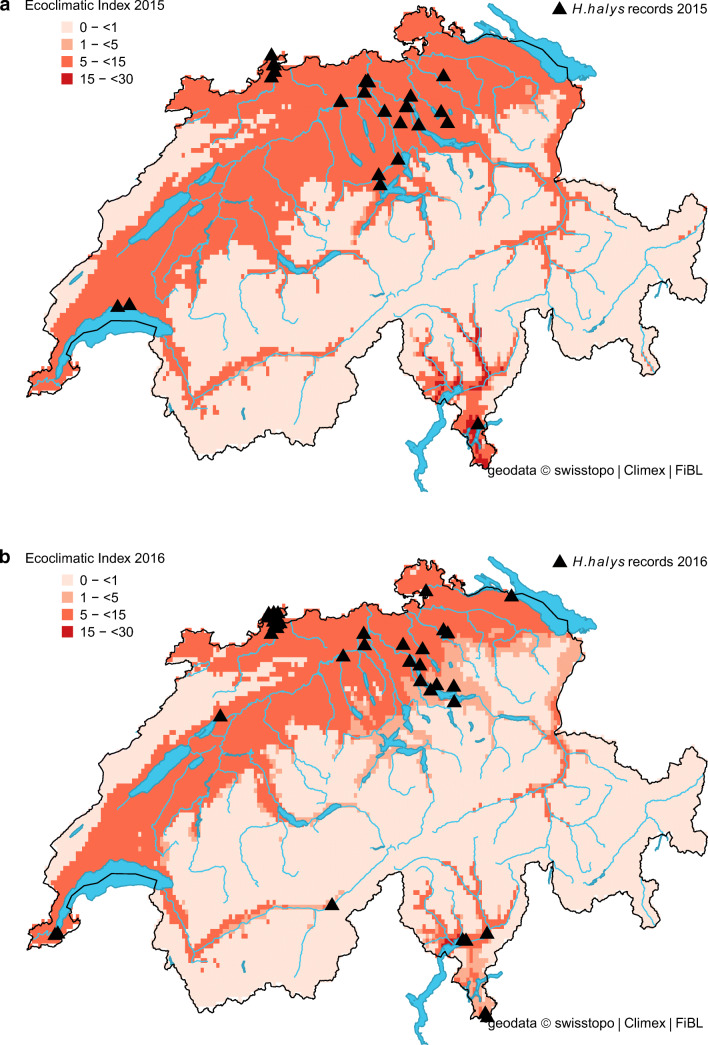

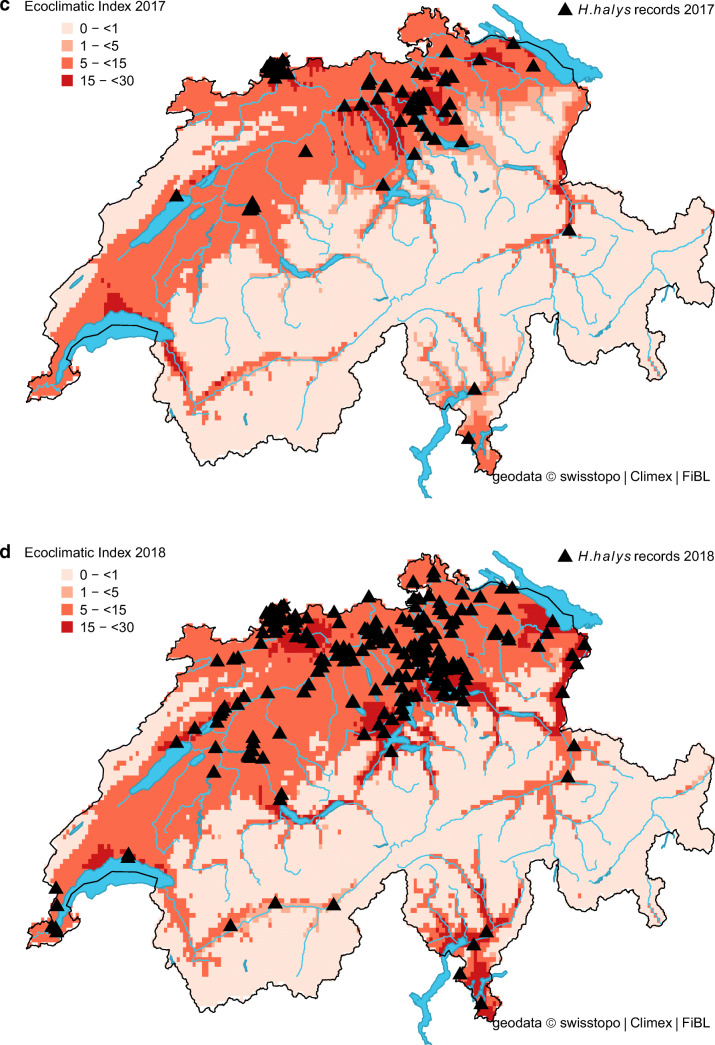

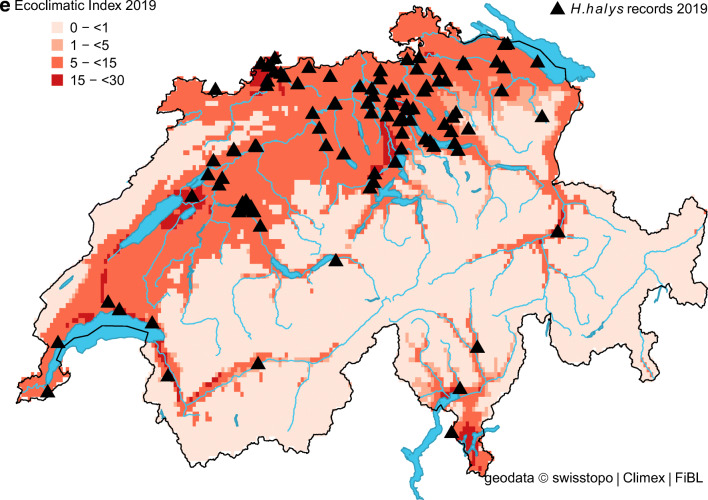
Modelled climate suitability (CLIMEX Ecoclimatic Index EI) for *Halyomorpha halys* in Switzerland for **a** 2015, **b** 2016, **c** 2017, **d** 2018, and **e** 2019. Black triangles represent observation records from 2015 to 2019

The projected distribution of *H. halys* in Switzerland matched well with its observed distribution (Fig. 1). In the beginning, most observations were recorded from the cities of Zurich, Basel, Bern and Lugano, but in recent years (2017–2019), observation reports from other areas noticeably increased and confirmed the presence of *H. halys* throughout the Swiss midlands (Fig. 2a–e). For the same period, our model indicated a remarkable increase in the proportion of suitable areas (Table 2). More specifically, it increased from 23.0% (reference period 1981–2010) to 49.1% in 2018, which corresponds with the highest number of observation records (Table 2 and Fig. 2d). The percentage of highly suitable area in 2018 was 8.4% compared to 0.3% for the reference period. Under present climate conditions, the model simulations estimated no cold, heat, wet or dry stress for any location in Switzerland, except Brig (canton of Valais), where *H. halys* seemed to have suffered from weak dry stress in 2016 and 2018. Between 33.2% (reference) and 50.1% (2018) of the country was suitable to support one (or more) *H. halys* generations (Table 2). The model indicated that in the period from 1981 to 2010, less than 1% of the country was able to support two *H. halys* generations per year. In contrast, this proportion increased to 6% in 2018 (Table 2). This finding illustrates the extended growing season in 2018 with a warm winter (2017 /18) followed by an exceptional warm spring, allowing the development of two *H. halys* generations in 2018.

### Potential distribution, phenology and voltinism under present and future climate conditions in Switzerland

**Fig. 3:**
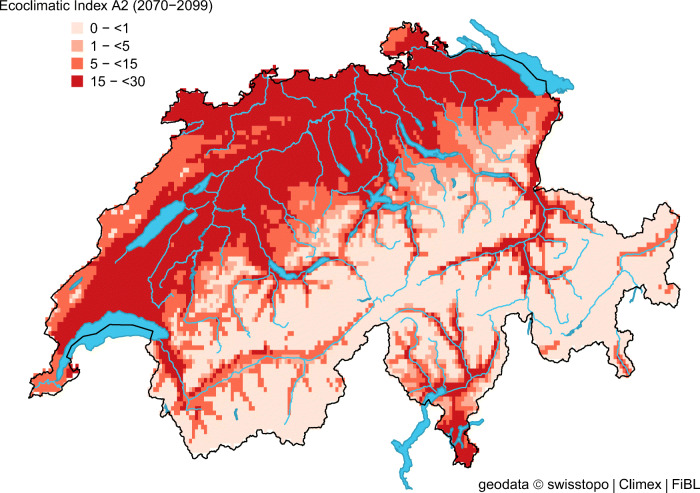
Modelled climate suitability (CLIMEX Ecoclimatic Index EI) for *Halyomorpha halys* in Switzerland for the A2 climate scenario (2070–2099) (see Fig. 1 for reference)

Compared to the reference period 1981–2010 (Fig. 1), the CLIMEX model projects an extensive expansion of the potential distribution in Switzerland for the A2 scenario (2070–2099) (Fig. 3). The north-western part of Switzerland could become completely suitable for *H. halys*. Southwards, the projected range expansion would reach the foothills of the Alps, and higher latitudes in the alpine valleys could become suitable under future climate conditions.

The A2 model output indicates a potential 2.7-fold increase of the percentage of suitable areas (EI > 5) by 2099 based on the reference period (Table 2). Even for the mitigation scenario RCP3PD, a 1.6-fold increase was simulated. Specifically our simulations illustrate that approximately 40–60% of Switzerland will be suitable for *H. halys* long-term survival by the end of the century. The increase of suitable areas in Switzerland between 2020 and 2049 is similar for all climate scenarios (Table 2). However, between 2045–2074 and 2070–2099, the percentage of suitable areas under the RCP3PD mitigation scenario remains low compared to the A1B and A2 scenario. With the A2 scenario, the model projects a massive increase in the percentage of the area with a high EI (EI > 15): from 0.3% (reference) to 36.9% (A2) (Table 2). There is no indication of cold, heat, wet or dry stress accrued under any of the climate scenarios (not presented). In Switzerland, the only factor leading to unfavourable conditions (EI = 0) is the relatively high number of degree days (595) required by *H. halys* to complete a generation.

For the reference period, we simulated that 33.2% of Switzerland would be suitable to support one or more generation of *H. halys* compared to 63.5% under the A2 climate change scenario (Table 2). Until only the last 2–3 years, two generations are only possible in exceptional years with unusual warm spring and summer temperatures (Table 2). By 2099, the A2 scenario projects two generations per year in 34.8% of the country compared to <1% for the reference period.

**Fig. 4:**
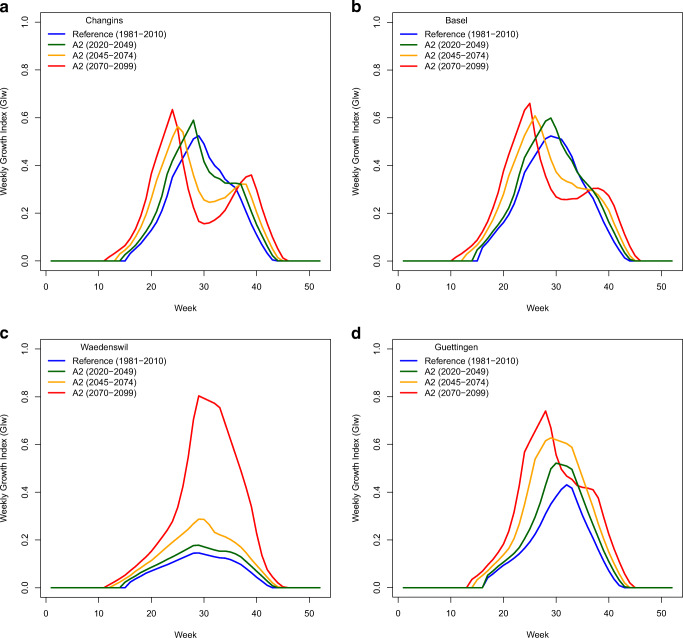
Weekly Growth Index GI_W_ for the reference (1981–2010) and the A2 scenario (time periods 2020–20459, 2045–2074, 2070–2099) at the four Swiss locations **a** Changins, **b** Basel, **c** Wädenswil, **d** Güttingen

A longer growing season under future climate scenarios is also visualised by the GI_W_ (Fig. 4) for four different locations, chosen because they represent four typical climate regions in Switzerland. Spring activity of the bugs is projected to start 1–4 weeks earlier compared to the reference period, depending on the climate scenario and time period. Similarly, the fall activity would stop 1–3 weeks later compared to the reference period. Although overall heat and dry stress under future climate scenarios may not affect the suitability, during some weeks high summer temperatures may exceed the limiting upper temperature threshold (DV3 = 33 °C). Consequently, for many locations in the southern and north-western parts of Switzerland (Fig. 4a, b), the projections indicate a decrease in the GI_W_ during the hot summer months (weeks 25–35), especially for the time period 2070–2099. For other locations, mainly in the north-eastern part of Switzerland, the growth potential would increase from 2020 to 2099 (Fig. 4c, d).

The projections for 10 representative locations in Switzerland show the highly variable impact of climate change on the EI, number of generations and number of weeks with positive GI_W_ (Table 3). According to the model, at nine out of ten locations the EI is expected to increase. Only in one location (Sion) a potential decrease is indicated due to high summer temperatures limiting the growth potential. Remarkably, under future climate conditions, all localities have the potential to host two or more *H. halys* generations per year, with the number of weeks of growth potential (GI_W_ > 0) projected to increase by 5–9 weeks (Table 3).

**Table 3:** Modelled climate suitability (CLIMEX Ecoclimatic Index, EI), number of generations and number of weeks with CLIMEX Growth Index, GI) > 0 for the reference period (1981–2010) and the three climate scenarios RCP3PD, A1B and A2 (2070–2099) at 10 Swiss localities

Location	Zurich	St. Gallen	Chur	Lugano	Sion	Lausanne	Delémont	Basel	Aarau	Bern
Latitude	47.38°	47.43°	46.85°	46.02°	46.23°	46.54°	47.37°	47.55°	47.39°	46.95°
Longitude	8.52°	9.39°	9.52°	8.95°	7.35°	6.58°	7.34°	7.57°	8.05°	7.44°
Altitude	408 m	674 m	593 m	275 m	515 m	495 m	435 m	260 m	384 m	542 m
Ecoclimatic Index (EI)										
Reference	7	0	14	15	5	14	12	16	12	10
A2	23	9	20	23	4	22	19	18	20	18
Number of generations										
Reference	1.35	0.91	1.32	1.83	1.63	1.53	1.35	1.55	1.35	1.14
A2	2.49	1.95	2.52	3.24	2.99	2.79	2.46	2.73	2.48	2.21
Number of weeks GI_W_ > 0										
Reference	26	25	26	31	26	30	27	29	27	25
A2	33	32	35	39	32	36	32	36	33	31

## Discussion

The validation of the CLIMEX model using the observational records collected in a citizen science initiative revealed that more than 15 years after its accidental introduction, *H. halys* has colonised nearly all bioclimatically suitable areas in Switzerland. Accordingly, there is no strong potential for range expansion into new areas in Switzerland under present climate conditions. By using local Swiss climate data with a spatial resolution of 0.02° (approx. 2 km), we identified many more climatically suitable areas for *H. halys* in Switzerland in comparison to previous CLIMEX models with a lower resolution (Kistner 2017; Kriticos et al. 2017). As suggested by Kriticos and Leriche (2010), our study demonstrates the increase in explanatory power when projecting a species niche model to climate data of higher resolution. For future research, we recommend examining the goodness of fit of other mechanistic models that also consider factors such as seasonal abundance of *H. halys*, diversity of agricultural landscapes, or presence of preferred host plants, which are not reflected in CLIMEX models. In addition, most CLIMEX models use standardised (average) species values, but such a simplification ignores *intraspecific* (within-*species*) *variation* that can be as extreme as the trait variation across species.

Our results are in line with the projection that the global potential range of *H. halys* will expand polewards and contract from its (warmer) southern range limits under future climate scenarios (Kistner 2017). The estimated climate suitability of *H. halys* in Switzerland under future climate scenarios suggests an extensive range expansion into higher altitudes of mountain regions (Alpine valleys, Swiss Jura Mountains) that are currently too cold for establishment. The percentage of area suitable for *H. halys* (EI > 5) could more than double under the more extreme climate change scenario by 2099 (A2, Table 2). Remarkably, we identified a pronounced increase in the proportion of highly suitable area (EI > 15) from 0.3% (reference) to 36.9% for the A2 climate scenario until the end of the century. More worryingly, climate change is projected to increase the number of weeks with positive GI_W_ (Table 3 and Fig. 4), resulting in earlier *H. halys* activity in spring and a prolonged period for nymphs to complete development in autumn. This in turn increases the likelihood that in most years, *H. halys* will have two instead of one generation, as already observed in the unusual warm years 2018 and 2019. These observations were underlined by the simulated increase of the area with two generations from 0.3% (reference) to 34.8% (A2) until the end of the century. However, for some locations, 3–4 generations per year are projected. This may be an overestimation of the number of annual generations due to the parameters used for the current CLIMEX model (Tab. 1), which is based on 595 degree days above the critical temperature threshold (*T*_0_) of 12 °C per generation (PDD). However, literature values for PDD and *T*_0_ are quite variable, ranging from 471 to 649 degree days (dd) and 11.1 to 13.9 °C (Haye et al. 2014; Musolin et al. 2019; Nielsen et al. 2008). In addition, the preoviposition period of overwintering *H. halys* females (148 dd) (Nielsen et al. 2008) is not reflected in the PDD. Another factor that may have influenced the model output is that the accuracy of the local climate data is not the same for all Swiss regions, e.g. for the temperature, an imprecision of 0.6 °C (midlands), 1.0 (alps) and 1.2 (Ticino) has been indicated (www.meteoschweiz.ch). Furthermore, we used the CH2011 climate scenarios based on 30-year mean temperature and precipitation changes, which does not account for potential changes in interannual variability, changes in wet-day frequency or dry-spell length.

Crop damage by *H. halys* in Switzerland was first reported in 2012 in greenhouse vegetables (Sauer 2012), and since 2016, damage in other crops such as pears, apples and grapes have steadily increased. Accordingly, a national monitoring programme was established in 2018 with the aim to better understand the distribution and seasonal phenology of *H. halys* in Switzerland. However, since *H. halys* was considered to still be spreading, the selection of monitoring locations was subject to uncertainty, and thus, the current model was developed to identify agricultural areas that are at higher risks to *H. halys* under current and future climates.

Our model projections suggest that the future increase in climate suitability and the expected expansion of the activity periods in spring and late autumn will likely result in a significant population growth of *H. halys*. Whereas warm temperatures in early spring would enable *H. halys* to develop two generations per year, unusual warm temperatures late in the season would allow more nymphs to complete development (Haye et al. 2014). Our model suggests that after 10 years of low population growth, such a scenario already occurred in the exceptional warm year of 2018. Consequently, a permanent shift from one to two generations per year and the associated population growth of *H. halys* would result in increasing crop damages in Switzerland, particularly in northern Switzerland and the Swiss midlands. Monitoring the spread and population development in the north-western part of Switzerland and higher altitudes of the valleys in the south, where new establishment of *H. halys* may occur in the near future, are recommended to protect threatened crops in a timely manner, using a variety of practices such as exclusion netting, bagging fruits, chemical or biological control (Candian et al. 2018; Lee et al. 2013; Leskey and Nielsen 2018).

The knowledge on the biology and ecology of *H. halys* is quickly growing and may help to further enhance the current model in the near future. Ideally, the precision of projecting species’ distribution and seasonal phenology should not only consider high-resolution climate data, but also habitat factors. For example, Kriticos et al. (2015a) demonstrated that including such factors resulted in a 22–53% reduction in the areas estimated to be endangered by the invasive plant species, *Parthenium hysterophorus* (Asteracae). Increasing temperatures may also lead to an increase in crop water demand resulting in an expanding area requiring irrigation during summer months, especially in southern and central Europe (EEA 2017; Wada et al. 2013). For the invasive wasp, *Vespula germanica* (Hymenoptera: Vespidae), it was shown that including an irrigation scenario into CLIMEX significantly improved the model fit between present and estimated distribution (de Villiers et al. 2017). Similarly, the use of irrigation systems may reduce the potential dry stress of *H. halys* during summer months, particularly in southern Switzerland, where irrigation is common practise. This may explain the observations records of *H. halys* in locations, where our simulations indicate a season-long dry stress However, for a polyphagous insect like *H. halys*, a complex modelling effort would be needed to include local and crop specific irrigation levels with daily resolution, differentiated timing and different amounts of irrigation, especially for countries with a complex landscape like Switzerland. Haye et al. (2014) showed that overwintering mortality of *H. halys* adults in an open wooden shelter in Switzerland was relatively low (39 %). Although temperature refugia provided by human-built structures can be crucial for overwintering survival in some areas (Cira et al. 2016), we do not consider overwintering mortality a major limiting factor for the distribution of *H. halys* in Switzerland.

Many CLIMEX models have focused on simulating the distribution of invasive pests, but only a few investigated the impact of climate change on the distribution of their antagonists (Haye et al. 2018; Olfert et al. 2016). Currently, there are no sufficient tools available for managing *H. halys* in Switzerland, but a very promising and sustainable approach could be the use of exotic natural enemies. The Asian samurai wasp, *Trissolcus japonicus* (Hymenoptera: Scelinoidae), has been identified as the most promising classical biological control agent in Asia and adventive populations were recently found in the Switzerland and Italy (Haye et al. 2020; Moraglio et al. 2020; Sabbatini Peverieri et al. 2018; Stahl et al. 2019). It remains unknown though, which areas will be most suitable for the establishment of the parasitoid in Switzerland and how climate change may alter its distribution. Similar to the current study, evaluating the bioclimatic suitability of *T. japonicus*, using the CLIMEX model of Avila and Charles (2018) and fine-scale climate data from Switzerland, could help to project the performance of the biological agent and identify optimal areas for its potential application.

## References

[CR1] Altermatt F (2010). Climatic warming increases voltinism in European butterflies and moths. Proc Roy Soc: Biol Sci.

[CR2] Avila GA, Charles JG (2018). Modelling the potential geographic distribution of *Trissolcus japonicus*: a biological control agent of the brown marmorated stink bug, *Halyomorpha halys*. BioControl.

[CR3] Bacon SJ, Aebi A, Calanca P, Bacher S (2013). Quarantine arthropod invasions in Europe: the role of climate, hosts and propagule pressure. Divers Distrib.

[CR4] Battisti DS, Naylor RL (2009). Historical warnings of future food insecurity with unprecedented seasonal heat. Science.

[CR5] Bebber DP, Ramotowski MAT, Gurr SJ (2013). Crop pests and pathogens move polewards in a warming world. Nat Clim Change.

[CR6] Bosco L, Moraglio ST, Tavella L (2018). *Halyomorpha halys*, a serious threat for hazelnut in newly invaded areas. J Pest Sci.

[CR7] Both C, van Asch M, Bijlsma RG, van den Burg AB, Visser ME (2009). Climate change and unequal phenological changes across four trophic levels: Constraints or adaptations?. J Anim Ecol.

[CR8] Byeon DH, Jung JM, Jung S, Lee WH (2018). Prediction of global geographic distribution of *Metcalfa pruinosa* using CLIMEX. Entomol Res.

[CR9] Caffarra A, Rinaldi M, Eccel E, Rossi V, Pertot I (2012). Modelling the impact of climate change on the interaction between grapevine and its pests and pathogens: European grapevine moth and powdery mildew. Agric Ecosyst Environ.

[CR10] Candian V, Pansa MG, Briano R, Peano C, Tedeschi R, Tavella L (2018). Exclusion nets: a promising tool to prevent *Halyomorpha halys* from damaging nectarines and apples in NW Italy. Bull Insectol.

[CR11] CH2011 (2011) Swiss Climate Change Scenarios CH2011. C2SM, MeteoSwiss, ETH, NCCR Climate, OcCC, Zurich, Switzerland

[CR12] CH2014-Impacts (2014) Toward quantitative scenarios of climate change impacts in Switzerland. OCCR, FOEN, MeteoSwiss, C2SM, Agroscope, and ProClim, Bern, Switzerland

[CR13] Cianferoni F, Graziani F, Dioli P, Ceccolini F (2018). Review of the occurrence of *Halyomorpha halys* (Hemiptera: Heteroptera: Pentatomidae) in Italy, with an update of its European and World distribution. Biologia.

[CR14] Cira TM, Venette RC, Aigner J, Kuhar T, Mullins DE, Gabbert SE, Hutchison WD (2016). Cold tolerance of *Halyomorpha halys* (Hemiptera: Pentatomidae) across geographic and temporal scales. Environ Entomol.

[CR15] Costi E, Haye T, Maistrello L (2017). Biological parameters of the invasive brown marmorated stink bug, *Halyomorpha halys*, in southern Europe. J Pest Sci.

[CR16] de Villiers M, Hattingh V, Kriticos DJ, Brunel S, Vayssieres JF, Sinzogan A, Billah MK, Mohamed SA, Mwatawala M, Abdelgader H, Salah FEE, De Meyer M (2016). The potential distribution of *Bactrocera dorsalis*: considering phenology and irrigation patterns. Bull Entomol Res.

[CR17] de Villiers M, Kriticos DJ, Veldtman R (2017). Including irrigation in niche modelling of the invasive wasp *Vespula germanica* (Fabricius) improves model fit to predict potential for further spread. PLoS One.

[CR18] Deutsch CA, Tewksbury JJ, Tigchelaar M, Battisti DS, Merrill SC, Huey RB, Naylor RL (2018). Increase in crop losses to insect pests in a warming climate. Science.

[CR19] Devictor V, van Swaay C, Brereton T, Brotons L, Chamberlain D, Heliola J, Herrando S, Julliard R, Kuussaari M, Lindstrom A, Reif J, Roy DB, Schweiger O, Settele J, Stefanescu C, Van Strien A, Van Turnhout C, Vermouzek Z, Wallis de Vries M, Wynhoff I, Jiguet F (2012). Differences in the climatic debts of birds and butterflies at a continental scale. Nat Clim Change.

[CR20] EEA (2017). Climate Change, Impacts and Vulnerability in Europe 2016: An indicator-based report.

[CR21] Felber R, Stoeckli S, Calanca P (2018). Generic calibration of a simple model of diurnal temperature variations for spatial analysis of accumulated degree-days. Int J Biometeorol.

[CR22] Gutierrez AP, Ponti L, Gilioli G, Rosenzweig C (2010). Climate change effects on plant-pest-natural enemy interactions. Handbook of Climate Change and Agroecosystems: Impacts, Adaptation, and Mitigation.

[CR23] Hance T, van Baaren J, Vernon P, Boivin G (2007). Impact of extreme temperatures on parasitoids in a climate change perspective. Annu Rev Entomol.

[CR24] Harrington R, Clark SJ, Welham SJ, Verrier PJ, Denholm CH, Hulle M, Maurice D, Rounsevell MD, Cocu N (2007). Environmental change and the phenology of European aphids. Global Change Biol.

[CR25] Haye T, Abdallah S, Gariepy T, Wyniger D (2014). Phenology, life table analysis, and temperature requirements of the invasive brown marmorated stink bug, *Halyomorpha halys,* in Europe. J Pest Sci.

[CR26] Haye T, Gariepy T, Hoelmer K, Rossi JP, Streito JC, Tassus X, Desneux N (2015). Range expansion of the invasive brown marmorated stinkbug, *Halyomorpha halys*: an increasing threat to field, fruit and vegetable crops worldwide. J Pest Sci.

[CR27] Haye T, Olfert O, Weiss R, Mason PG, Gibson G, Gariepy TD, Gillespie DR (2018). Bioclimatic analyses of *Trichomalus perfectus* and *Mesopolobus morys* (Hymenoptera: Pteromalidae) distributions, two potential biological control agents of the cabbage seedpod weevil in North America. Biol Control.

[CR28] Haye T, Moraglio ST, Stahl J, Visentin S, Gregorio T, Tavella L (2020). Fundamental host range of *Trissolcus japonicus* in Europe. J Pest Sci.

[CR29] Henne PD, Bigalke M, Buntgen U, Colombaroli D, Conedera M, Feller U, Frank D, Fuhrer J, Grosjean M, Heiri O, Luterbacher J, Mestrot A, Rigling A, Rossler O, Rohr C, Rutishauser T, Schwikowski M, Stampfli A, Szidat S, Theurillat JP, Weingartner R, Wilcke W, Tinner W (2018). An empirical perspective for understanding climate change impacts in Switzerland. Reg Env Change.

[CR30] Hickling R, Roy DB, Hill JK, Fox R, Thomas CD (2006). The distributions of a wide range of taxonomic groups are expanding polewards. Global Change Biol.

[CR31] Hill MP, Thomson LJ, Björkman C, Niemalä P (2015). Species distribution modelling in predicting response to climate change. Climate Change and Insect Pests.

[CR32] Hoebeke ER, Carter ME (2003). *Halyomorpha halys* (Stal) (Heteroptera : Pentatomidae): A polyphagous plant pest from Asia newly detected in North America. Proc Entomol Soc Wash.

[CR33] Hofmann ME, Hinkel J, Wrobel M (2011). Classifying knowledge on climate change impacts, adaptation, and vulnerability in Europe for informing adaptation research and decision-making: a conceptual meta-analysis. Glob Environ Change.

[CR34] Jactel H, Koricheva J, Castagneyrol B (2019). Responses of forest insect pests to climate change: not so simple. Cur Opi Insect Sci.

[CR35] Jasper K, Calanca P, Gyalistras D, Fuhrer J (2004). Differential impacts of climate change on the hydrology of two alpine river basins. Clim Res.

[CR36] Jonsson AM, Appelberg G, Harding S, Barring L (2009). Spatio-temporal impact of climate change on the activity and voltinism of the spruce bark beetle, *Ips typographus*. Global Change Biol.

[CR37] Kistner EJ (2017). Climate change impacts on the potential distribution and abundance of the brown marmorated stink bug (Hemiptera: Pentatomidae) with special reference to North America and Europe. Environ Entomol.

[CR38] Kistner-Thomas EJ (2019) The potential global distribution and voltinism of the Japanese beetle (Coleoptera: Scarabaeidae) under current and future climates. J Insect Sci 19(2). 10.1093/jisesa/iez023 10.1093/jisesa/iez023 PMC6429693 30900722

[CR39] Kocmankova E, Trnka M, Eitzinger J, Dubrovsky M, Stepanek P, Semeradova D, Balek J, Skalak P, Farda A, Juroch J, Zalud Z (2011). Estimating the impact of climate change on the occurrence of selected pests at a high spatial resolution: A novel approach. J Agric Sci.

[CR40] Kriticos DJ, Leriche A (2010). The effects of climate data precision on fitting and projecting species niche models. Ecography.

[CR41] Kriticos DJ, Webber BL, Leriche A, Ota N, Macadam I, Bathols J, Scott JK (2012). CliMond: global high-resolution historical and future scenario climate surfaces for bioclimatic modelling. Methods Ecol Evol.

[CR42] Kriticos DJ, Brunel S, Ota N, Fried G, Lansink A, Panetta FD, Prasad TVR, Shabbir A, Yaacoby T (2015). Downscaling pest risk analyses: identifying current and future potentially suitable habitats for *Parthenium hysterophorus* with particular reference to Europe and North Africa. PLoS One.

[CR43] Kriticos DJ, Maywald GF, Yonow T, Zurcher EJ, Hermann NI, Sutherst RW (2015). CLIMEX Version 4: exploring the effects of climate on plants, animals and diseaes.

[CR44] Kriticos DJ, Ota N, Hutchison WD, Beddow J, Walsh T, Tay WT, Borchert DM, Paula-Moreas SV, Czepak C, Zalucki MP (2015). The potential distribution of invading *Helicoverpa armigera* in North America: is it just a matter of time?. PLoS One.

[CR45] Kriticos DJ, Kean JM, Phillips CB, Senay SD, Acosta H, Haye T (2017). The potential global distribution of the brown marmorated stink bug, *Halyomorpha halys*, a critical threat to plant biosecurity. J Pest Sci.

[CR46] Langille AB, Arteca EM, Newman JA (2017) The impacts of climate change on the abundance and distribution of the spotted wing drosophila (*Drosophila suzukii*) in the United States and Canada. Peerj 5. 10.7717/peerj.3192 10.7717/peerj.3192 PMC5385127 28396828

[CR47] Lee DH, Short BD, Joseph SV, Bergh JC, Leskey TC (2013). Review of the biology, ecology, and management of *Halyomorpha halys* (Hemiptera: Pentatomidae) in China, Japan, and the Republic of Korea. Environ Entomol.

[CR48] Leskey TC, Nielsen AL (2018). Impact of the invasive brown marmorated stink bug in North America and Europe: history, biology, ecology, and management. Annu Rev Entomol.

[CR49] Leskey TC, Short BD, Butler BR, Wright SE (2012). Impact of the invasive brown marmorated stink bug, *Halyomorpha halys*, in Mid-Atlantic tree fruit orchards in the United States: case studies of commercial management. Psyche J Ent.

[CR50] Maclean IMD, Wilson RJ (2011). Recent ecological responses to climate change support predictions of high extinction risk. Proc Natl Acad Sci U S A.

[CR51] Maistrello L, Vaccari G, Caruso S, Costi E, Bortolini S, Macavei L, Foca G, Ulrici A, Bortolotti PP, Nannini R, Casoli L, Fornaciari M, Mazzoli GL, Dioli P (2017). Monitoring of the invasive *Halyomorpha halys*, a new key pest of fruit orchards in northern Italy. J Pest Sci.

[CR52] Meynard CN, Migeon A, Navajas M (2013). Uncertainties in predicting species distributions under climate change: a case study using *Tetranychus evansi* (Acari: Tetranychidae), a widespread agricultural pest. PLoS One.

[CR53] Moraglio ST, Tortorici F, Pansa MG, Castelli G, Pontini M, Scovero S, Visentin S, Tavella L (2020). A 3-year survey on parasitism of *Halyomorpha halys* by egg parasitoids in northern Italy. J Pest Sci.

[CR54] Musolin DL, Dolgovskaya MY, Protsenko VY, Karpun NN, Reznik SY, Saulich AK (2019). Photoperiodic and temperature control of nymphal growth and adult diapause induction in the invasive Caucasian population of the brown marmorated stink bug, *Halyomorpha halys*. J Pest Sci.

[CR55] Nielsen AL, Hamilton GC, Matadha D (2008). Developmental rate estimation and life table analysis for *Halyomorpha halys* (Hemiptera : Pentatomidae). Environ Entomol.

[CR56] Oerke EC (2006). Crop losses to pests. J Agric Sci.

[CR57] Olfert O, Haye T, Weiss R, Kriticos D, Kuhlmann U (2016). Modelling the potential impact of climate change on future spatial and temporal patterns of biological control agents: *Peristenus digoneutis* (Hymenoptera: Braconidae) as a case study. Can Entomol.

[CR58] Parmesan C, Yohe G (2003). A globally coherent fingerprint of climate change impacts across natural systems. Nature.

[CR59] Porter JH, Parry ML, Carter TR (1991). The potential effects of climate change on agricultural insect pests. Agricultural and Forest Meteorology.

[CR60] Ramos RS, Kumar L, Shabani F, da Silva RS, de Araujo TA, Picanco MC (2019). Climate model for seasonal variation in *Bemisia tabaci* using CLIMEX in tomato crops. Int J Biometeorol.

[CR61] Robinet C, Roques A (2010). Direct impacts of recent climate warming on insect populations. Integr Zool.

[CR62] Roth T, Plattner M, Amrhein V (2014) Plants, birds and butterflies: Short-term responses of species communities to climate warming vary by taxon and with altitude. PLoS One 9 (1):e82490. doi:82410.81371/journal.pone.0082490 10.1371/journal.pone.0082490 PMC3885385 24416144

[CR63] Sabbatini Peverieri G, Binazzi F, Marianelli L, Roversi PF (2018). Lethal and sublethal effects of long-lasting insecticide-treated nets on the invasive bug *Halyomorpha halys*. J Appl Entomol.

[CR64] Sauer C (2012). Die Marmorierte Baumwanze tritt neu im Deutschschweizer Gemüsebau auf. Gemüsebau Info.

[CR65] Seebens H, Blackburn TM, Dyer EE, Genovesi P, Hulme PE, Jeschke JM, Pagad S, Pysek P, Winter M, Arianoutsou M, Bacher S, Blasius B, Brundu G, Capinha C, Celesti-Grapow L, Dawson W, Dullinger S, Fuentes N, Jager H, Kartesz J, Kenis M, Kreft H, Kuhn I, Lenzner B, Liebhold A, Mosena A, Moser D, Nishino M, Pearman D, Pergl J, Rabitsch W, Rojas-Sandoval J, Roques A, Rorke S, Rossinelli S, Roy HE, Scalera R, Schindler S, Stajerova K, Tokarska-Guzik B, van Kleunen M, Walker K, Weigelt P, Yamanaka T, Essl F (2017). No saturation in the accumulation of alien species worldwide. Nat Commun.

[CR66] Stahl J, Tortorici F, Pontini M, Bon M-C, Hoelmer K, Marazzi C, Tavella L, Haye T (2019). First discovery of adventive populations of *Trissolcus japonicus* in Europe. J Pest Sci.

[CR67] Stoeckli S, Hirschi M, Spirig C, Calanca P, Rotach MW, Samietz J (2012) Impact of climate change on voltinism and prospective diapause induction of a global pest insect - *Cydia pomonella* (L.). PLoS One 7 (4):e435723. doi:435710.431371/journal.pone.0035723 10.1371/journal.pone.0035723 PMC3335082 22539997

[CR68] Thomson LJ, Macfadyen S, Hoffmann AA (2010). Predicting the effects of climate change on natural enemies of agricultural pests. Biol Control.

[CR69] Trnka M, Muska F, Semeradova D, Dubrovsky M, Kocmankova E, Zalud Z (2007). European Corn Borer life stage model: regional estimates of pest development and spatial distribution under present and future climate. Ecol Model.

[CR70] Tylianakis JM, Didham RK, Bascompte J, Wardle DA (2008). Global change and species interactions in terrestrial ecosystems. Ecol Lett.

[CR71] Tytar VM, Kozynenko II (2020). Bioclimatic modeling of the distribution of brown marmorated stink bug *Halyomorpha halys* (St&aring;l, 1855), with special reference to Ukraine. Dopov Nac akad nauk Ukr.

[CR72] Vera MT, Rodriguez R, Segura DF, Cladera JL, Sutherst RW (2002). Potential geographical distribution of the Mediterranean fruit fly, *Ceratitis capitata* (Diptera : Tephritidae), with emphasis on Argentina and Australia. Environ Entomol.

[CR73] Wada Y, Wisser D, Eisner S, Florke M, Gerten D, Haddeland I, Hanasaki N, Masaki Y, Portmann FT, Stacke T, Tessler Z, Schewe J (2013). Multimodel projections and uncertainties of irrigation water demand under climate change. Geophys Res Lett.

[CR74] Walther GR, Post E, Convey P, Menzel A, Parmesan C, Beebee TJC, Fromentin JM, Hoegh-Guldberg O, Bairlein F (2002). Ecological responses to recent climate change. Nature.

[CR75] Warren R, Price J, Graham E, Forstenhaeusler N, Van der Wal J (2018). The projected effect on insects, vertebrates, and plants of limiting global warming to 1.5 °C rahter than 2 °C. Science.

[CR76] Wilby RL, Troni J, Biot Y, Tedd L, Hewitson BC, Smith DM, Sutton RT (2009). A review of climate risk information for adaptation and development planning. Int J Climatol.

[CR77] Yonow T, Kriticos DJ, Ota N, Van Den Berg J, Hutchison WD (2017). The potential global distribution of *Chilo partellus*, including consideration of irrigation and cropping patterns. J Pest Sci.

[CR78] Yudelman M, Ratta A, Nygaard D (1998) Pest management and food production. Looking to the future. International Food Policy Research Institute, Washington DC, USA

[CR79] Ziter C, Robinson EA, Newman JA (2012). Climate change and voltinism in Californian insect pest species: sensitivity to location, scenario and climate model choice. Global Change Biol.

[CR80] Zubler EM, Fischer AM, Liniger MA, Croci-Maspoli M, Scherrer SC, Appenzeller C (2014). Localized climate change scenarios of mean temperature and precipitation over Switzerland. Clim Change.

